# Comparative Analysis of Solvent Casting and Pickering Emulsion Techniques for Improving the Mechanical Properties of Surface-Modified Cellulose Nanomaterial-Reinforced Polylactic Acid Composites

**DOI:** 10.3390/polym16233406

**Published:** 2024-12-03

**Authors:** Faik Bolat, Madalina Ioana Necolau, Elena Iuliana Bîru, Anamaria Zaharia, Horia Iovu

**Affiliations:** 1 Advanced Polymer Materials Group, National University of Science and Technology Politehnica Bucharest, 1–7 Gh. Polizu Street, 011061 Bucharest, Romania; 2 Academy of Romanian Scientists, Ilfov 3, 050044 Bucharest, Romania; 3 Advanced Polymer Materials and Polymer Recycling Group, National Institute for Research and Development in Chemistry and Petrochemistry-ICECHIM, Splaiul Independenței 202, 060021 Bucharest, Romania

**Keywords:** polylactic acid (PLA), cellulose nanomaterials, Pickering emulsion, solvent casting, mechanical properties, surface modification, biodegradable composites, sustainable packaging, 2,4-methylene diphenyl diisocyanate (MDI), castor oil

## Abstract

In the present work, solvent casting and Pickering emulsion methods are studied to enhance the mechanical properties of polylactic acid (PLA) composites containing surface-modified cellulose nanomaterials. To enhance the compatibility and the adhesion at the interface, cellulose nanocrystal (CNC) was functionalized by 2,4-methylene diphenyl diisocyanate (MDI) and castor oil. Their results demonstrated that the Pickering emulsion method led to better dispersion of CNC in composites, resulting in improved tensile strength, flexibility, and thermal stability (compared with solvent-casted ones). In particular, the tensile strength increased by 20% and the crystallinity increased by 15% using the Pickering emulsion technique, indicating their potential as a new generation of sustainable packaging. The findings of this research could help in creating eco-friendly packaging options by improving the mechanical features of biodegradable composites and exploring potential alternatives to overcome their limitations.

## 1. Introduction

A grand challenge within the domain of materials science and engineering is to develop advanced materials with improved mechanical performance that also support sustainability. This issue is highly important when it comes to biodegradable packaging, which is crucial for addressing the environmental issues associated with conventional plastics. Regular plastic bags can remain in oceans for hundreds of years, playing a significant role in pollution that ultimately harms wildlife [1]. By focusing on materials that possess both mechanical strength and environmental sustainability, we can develop packaging solutions that reduce waste and decrease ecological damage.

In the packaging industry, the mechanical properties of polymers are critical in determining their suitability for specific applications. Table 1 summarizes key properties such as Young’s modulus, tensile strength, and elongation at break for both biopolymer-based plastics like poly(lactic acid) (PLA) and poly(hydroxyalkanoates) (PHAs), as well as traditional petrochemical-based plastics, including low-density polyethylene (LDPE), high-density polyethylene (HDPE), and poly(ethylene terephthalate) (PET). These values underline the mechanical advantages of conventional plastics, such as the high elongation of LDPE and HDPE, in contrast with the rigidity and lower ductility of biopolymers like PLA, highlighting the need for further improvement in the performance of biopolymers.

Biodegradable materials reduce waste and ecological impact by decomposing more quickly and safely. They ease the shift to a sustainable circular economy by using renewable resources and decreasing dependence on fossil fuels. Polylactic acid (PLA) is a renewable and biodegradable polymer made from corn starch or sugarcane. PLA has received considerable interest due to its eco-friendly features and its potential to serve as an alternative for traditional petroleum-based plastics across various applications. However, its natural brittleness and restricted mechanical strength have hindered its widespread use [5,6].

To overcome these limitations, researchers have investigated the addition of reinforcing agents and modifiers into the PLA matrix, intending to increase its mechanical properties while preserving biodegradability. One type of material that holds significant potential in this area is cellulose nanomaterials, such as cellulose nanocrystal (CNC) and cellulose nanofibril (CNF). These nanomaterials are derived from renewable resources like wood pulp and exhibit outstanding mechanical properties such as high tensile strength and stiffness. The combination of CNC and CNF with PLA matrices is expected to result in strengthened composites and providing them with higher stiffness, hence widening their utilization in more fields [7,8,9].

Recently, many experts from all over the world have shared numerous studies related to preparation methods, chemical structures, and applications of nanocellulose and nanocellulose composite materials [10,11,12]. Nanocellulose can be prepared by different methodologies, comprising mechanical processes, chemical treatments, enzymatic hydrolysis, etc., and hybrid approaches may be incorporated for maintaining the yield, quality, and environmental sustainability. The various studies carried out on the structural properties and performance characteristics of nanocellulose highlight its high crystallinity, functional surface characteristics, remarkable mechanical strength, thermal stability, barrier properties, and biodegradability, which all target a wide field of applications [12,13,14,15]. Biomedical and green packaging to flexible electronics and water purification are just a few examples of nanocellulose applications, while future studies will be oriented toward scalability, cost-effectiveness, and customization to enable further industrial utilization [16,17].

Surface modification plays a key role in improving the compatibility of cellulose nanomaterials with polymer matrices, such as PLA. Both CNC and CNF can be optimized for dispersion and interaction within a polymer matrix by applying appropriate modifications on their surface, which contributes to improved performance of the composite material. One such modification approach involves the use of 1,4 MDI or methylene diphenyl diisocyanate along with castor oil. The main reason for using 1,4 MDI in surface modification is due to its capacity to establish covalent bonds with hydroxyl groups present on the cellulose surface [18]. Due to the abundant hydroxyl groups, the surface of cellulose nanomaterials is hydrophilic in nature. This usually leads to inadequate compatibility between celluloses and hydrophobic polymer matrices. The incorporation of 1,4 MDI, which interacts with the hydroxyl groups to create urethane linkages, is expected to enhance the interfacial adhesion between cellulose and the polymer matrix [19]. Using 1,4 MDI may decrease the hydrophilicity of cellulose, which could lead to a decreased tendency for cellulose to clump together in a hydrophobic polymer matrix. This significantly improves cellulose nanomaterial dispersion within the polymer, which is a critical attribute for maximizing the reinforcement effect and maintaining uniform properties in composite materials. In this study, we aim to explore how the surface modification of cellulose nanomaterials with 2,4-MDI and castor oil affects the mechanical properties of PLA composite materials. In the MDI molecule, there are two NCO groups in ortho and para positions, and their reactivities are different. It can be considered that the spatial configuration of the isocyanate groups may affect its reactivity and the characteristics of the molecules. Normally, the ortho NCO group in 2,4-MDI shows relatively lower reactivity compared to the para-NCO group [20]. The para position allows the isocyanate group to experience less hinderance and, therefore, be more available toward chemical attack. A study indicated that the isocyanate group located in the ortho position was three times less reactive compared to the isocyanate in the para position [21]. On the other hand, this difference in reactivity between the two NCO groups can be useful while developing or changing any materials; it provides a higher level of control over the reaction process and the qualities of the final product. Castor oil provides plasticization and compatibilization, which enhances the flexibility of any composite material. Furthermore, detailed studies on differing concentrations of modified cellulose nanomaterials and their effect on the properties of the composite will be established in order to provide an optimum formulation for the enhanced mechanical performance of the composites.

The popularity of Pickering emulsions as a method for incorporating nanocellulose into composite materials has increased due to the amphiphilic nature of nanocellulose, which facilitates the effective stabilization of emulsions and improves compatibility with hydrophobic matrices. Similarly, advancements in pavement composition and preparation techniques have focused on enhancing the viscoelastic and adhesive properties of bitumen through the incorporation of innovative additives and nanomaterials, contributing to improved performance and durability [22].

The process of grafting 2,4-methylene diphenyl diisocyanate (MDI) onto the surface of cellulose nanomaterials represents a chemical modification technique that can improve the compatibility and reactivity of cellulose with a range of polymers. Grafting only one side of a 2,4-methylene diphenyl diisocyanate (MDI) molecule onto the surface of cellulose nanomaterials can be achieved through careful control of the reaction conditions. This process is commonly known as ‘mono-functionalization’ or ‘single-sided grafting’ [23,24].

## 2. Materials and Methods

### 2.1. Materials

Polylactic acid (PLA) pellets was purchased from Gembird (Wittevrouwen, The Netherlands), with a density of 1.25 g·cm^−3^ at 21.5 °C, melting point of 190–220 °C, heat-deflection-temperature of 0.455 MPa: 50 °C, average molecular weight between 60,000 and 160,000 g/mol, and polydispersity (PDI) between 1.5 and 2.0. Castor oil, derived from the Ricinus communis plant, is a commercially available product that consists of 90% ricinoleic acid and has a density of 950 kg/m^3^, purchased from the local market Herbavit (Oradea, Romania). Cellulose nanocrystal (nanocrystalline cellulose, CNC) in the form of powder was purchased from Nanography (Ankara, Turkey), (Average particle dimensions range from 10 to 20 nanometers in width and 300 to 900 nanometers in length, density, 1.49 g/cm^3^; cellulose crystallinity (XRD), 92%). 2,4’-methylene diphenyl diisocyanate (MDI), toluene, dichloromethane (DCM), and dibutyltin dilaurate 95% (DBTL) were used as received from Sigma Aldrich, Burlington, MA, USA.

### 2.2. Methods

#### 2.2.1. Grafting of MDI onto the CN Surface

**Figure 1:**
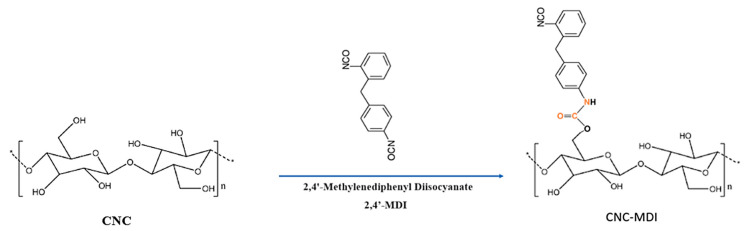
Reaction between CNC and MDI.

2 g of dried CNC was dispersed in 50 milliliters of anhydrous toluene and subjected to sonication at 50% amplitude for 10 min using a Sonics Viba cell Sonication device at 50% amplitude in the 20–40 kHz range in an ultrasound bath. Subsequently, 4 g of MDI were introduced into the reaction flask and the reaction was sustained at 80 °C for four hours under a nitrogen gas environment (Figure 1). After the reaction was terminated, the resulting mixture was centrifuged at 9000 rpm to separate the MDI-carbamated CNC (CNC-MDI) from any leftover unreacted MDI. The product was subsequently rinsed five times with dry toluene and then dried at 25 °C, resulting in 2.1 g of CNC-MDI.

#### 2.2.2. Grafting of NCO-Terminated CNC-MDI with Castor Oil

**Figure 2:**
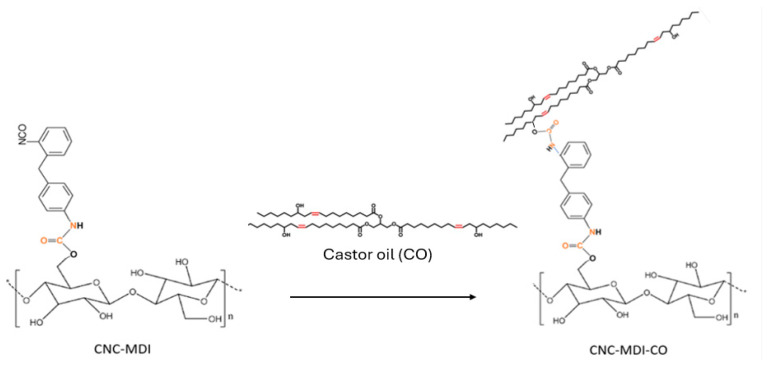
Reaction between modified CNC and Castor oil.

1.5 g of CNC-MDI was dissolved in 50 milliliters of anhydrous toluene and subjected to sonication at 50% amplitude for 10 min. Following the complete dispersion of the cellulose, 4.5 g of castor oil and 0.3 milliliters of DBTL were introduced; the reaction was sustained at 80 °C for 15 h. After the reaction was completed, the mixture was centrifuged to isolate the CNC-MDI-castor oil (CNC-MDI-CO) from any unreacted castor oil, followed by washing with toluene and subsequent drying. A schematic representation of the modification process is presented in Figure 2.

#### 2.2.3. PLA-CNC-Based Nanocomposite Synthesis

**Figure 3:**
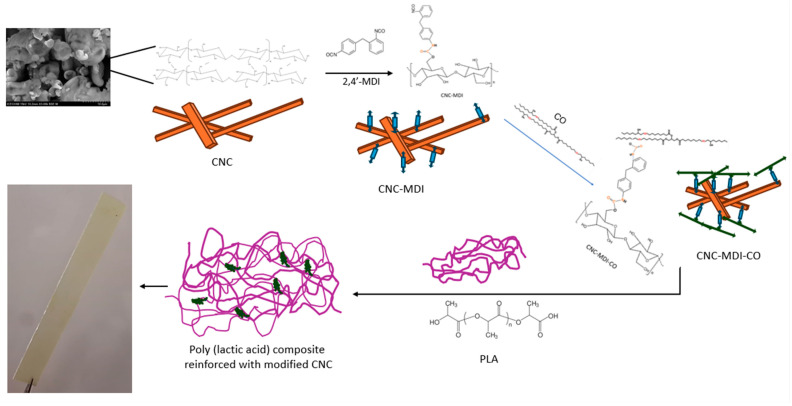
Schematic representation of CNC modification and PLA nanocomposite preparation.

Solvent casting and Pickering emulsion are two different methods that have been adopted to synthesize PLA nanocomposites based on CNC and CNC-MDI-CO, as shown in Figure 3.

In the solvent-casting technique, a substance is dissolved in a volatile solvent, followed by spreading the solution onto a substrate, which allows the formation of a solid film to occur by evaporation. The basic mechanism of Pickering emulsions is different; the process relies on the adsorption of solid particles at the boundary between two non-mixing liquid phases. Such adsorption creates a physical barrier that prevents droplet coalescence. Factors determining the stability of an emulsion include wettability, size, concentration, shape, and the surface characteristics of the particles, as well as pH and ionic strength [25].

**Figure 4:**
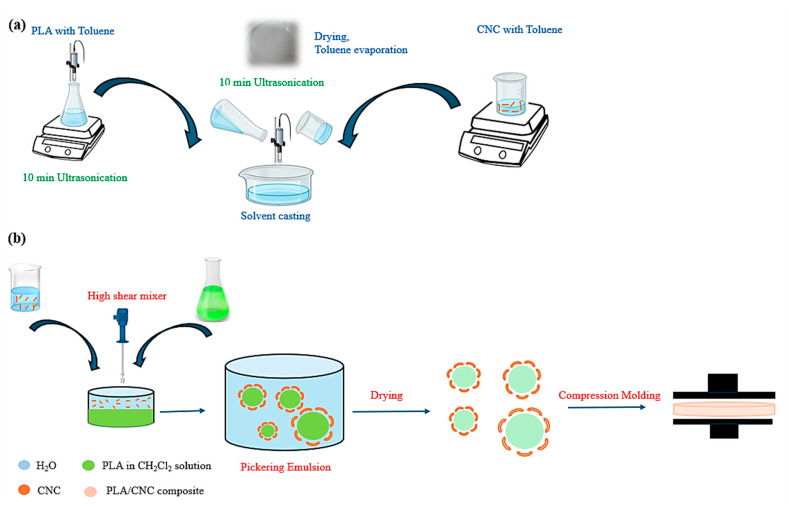
Schematic illustration of the production of PLA-CNC composite films through (**a**) solvent casting and (**b**) Pickering emulsion.

In the solvent-casting technique, in a container, 4 g of PLA was dissolved in 100 mL toluene and then, in a second container, varying amounts of CNC and CNC-MDI-CO were added at concentrations of 1, 3, and 5 wt.%, relative to the PLA weight, then the samples were dispersed in 25 mL toluene. Afterward, they were sonicated for 10 min in an ice bath. The two solutions were then mixed, followed by further sonication for 10 min and casting in a Petri dish; finally, the drying process was carried out at room temperature for 24 h, as illustrated in Figure 4a.

For the preparation of Pickering emulsions, 4 g of PLA was dissolved in 100 mL of dichloromethane (DCM) to create a uniform organic phase. The aqueous phase was prepared by dispersing cellulose nanocrystals and CNC modified with isocyanate in water. In this case, cellulose acts as a stabilizing agent at the interface between the organic phase (PLA in DCM) and the aqueous phase (water), effectively preventing the separation of these two phases. After the preparation of the two phases, mixing was performed at a shear rate of 2000 s⁻^1^ and a temperature of 25 °C for one hour before transferring into a Petri dish, allowing air drying at room temperature for 24 h. As DCM gradually evaporated from the Pickering emulsion stabilized by CNC, PLA/CNC composite microspheres were obtained. Finally, a uniformly distributed PLA/CNC composite was successfully produced through compression molding at a temperature of 180 °C, using a force of 15,000~20,000 N applied for 5 min, as schematically presented in Figure 4b. As a result of this process, the obtained PLA/CNC composite showed improved dispersion of the CNC particles when compared to the conventional solvent-casting method.

### 2.3. Characterization Techniques

#### 2.3.1. Fourier–Transformed Infrared Spectrometry (FTIR) Analysis

The chemical composition of the selected samples was analyzed through FTIR spectroscopy, utilizing a Vertex 70 Bruker FTIR spectrometer fitted with an attenuated total reflectance (ATR) accessory (Bruker, Billerica, MA, USA). For each sample, 32 scans were conducted in the ATR-FTIR mode, achieving a resolution of 4 cm^−1^ across the wavenumber range of 600–4000 cm^−1^.

#### 2.3.2. Scanning Electron Microscopy (SEM)

SEM analyses were performed using a Hitachi TM4000plus II tabletop scanning electron microscope (SEM) (Spectral, Lidingo, Sweden), equipped with a cooling stage and operated at 15 kV. To mitigate “charging”, reduce thermal damage, and improve secondary electron emission, a thin layer of electrically conductive gold was deposited on the samples before the SEM analysis.

#### 2.3.3. TGA Analysis

Thermogravimetric analysis (TGA) was performed using a Netzsch TG 209 F1 Libra instrument (Selb, Germany). Samples, approximately 3–5 mg in weight, were placed in a platinum crucible, and thermograms were recorded across a temperature range of 20 to 200 °C, with a heating rate established at 10 °C/min, all conducted under a nitrogen flow rate of 10 mL/min.

#### 2.3.4. Differential Scanning Calorimetry (DSC) Analysis

The calorimetric analysis was conducted utilizing a Netzsch DSC 204 F1 Phoenix differential scanning calorimeter (Selb, Germany), operating with a continuous nitrogen flow maintained at 20 mL/min. Samples of approximately 5 mg were positioned in aluminum pans, and the DSC thermograms were captured over a temperature range of 25 to 200 °C, employing a heating rate of 10 °C/min.

#### 2.3.5. Dynamic Mechanical Analysis (DMA)

Dynamic mechanical analysis (DMA) was performed using a TRITEC 2000 B apparatus produced by Triton Technology, Ltd. Now Metler Toledo (Greifensee, Switzerland) employing a heating rate of 5 °C per minute in single cantilever bending mode at a frequency of 1 Hz and a deformation amplitude of 20 µm across a temperature spectrum from room temperature to 120 °C.

#### 2.3.6. X-Ray Photoelectron Spectroscopy (XPS)

X-ray photoelectron spectroscopy (XPS) measurements were performed utilizing a Thermo Scientific K-Alpha instrument (Thermo Scientific, East Grinstead, UK), which employed a monochromatic Al Kα source (1486.6 eV) and maintained a pressure of 2 × 10^−9^ mbar. The binding energy calibration was achieved by referencing the C1s peak at 284.8 eV as the internal standard.

#### 2.3.7. Contact Angle (CA)

Contact angle measurements were performed using a Drop Shape Analyzer-DSA100 from Krüss Scientific GmbH (Hamburg, Germany), applying the static sessile drop method at room temperature. Deionized purified water and methylene iodide were utilized as reference polar and nonpolar liquids, respectively. A droplet of 2 μL was maintained on the sample for a period of 5 s. The water contact angle was determined through the Young–Laplace equation within the KRÜSS ADVANCE 1.7.2.1 software, with the reported value reflecting the average of three measurements for each sample.

#### 2.3.8. Mechanical Tests

Mechanical testing was conducted utilizing a universal mechanical tester, specifically the Instron Model 3382 (Instron, Model 3382, Norwood, MA, USA), under conditions of relative humidity ranging from 45 to 50% and at a testing speed of 1 mm/min. For each nanocomposite system, a minimum of three specimens, shaped as rectangular bars with dimensions of 125 mm (length) × 12.7 mm (width) × 3 mm (thickness), were evaluated, and the average results are presented.

## 3. Results and Discussion

### 3.1. FTIR Analysis of CNC and Modified CNC Samples

**Figure 5:**
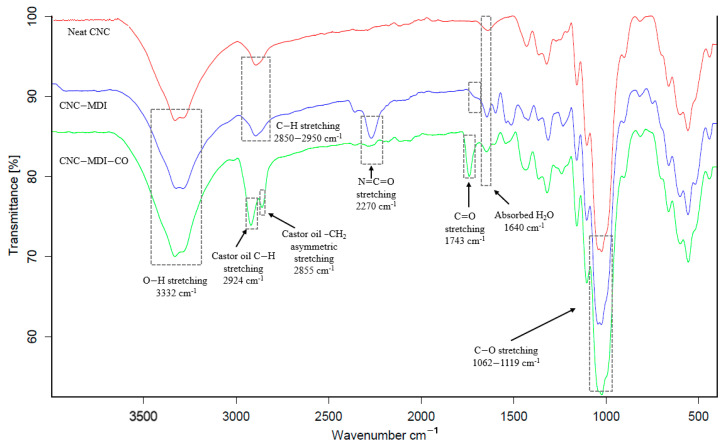
FTIR spectra of neat and modified CNC samples.

FTIR analysis was conducted to investigate the chemical structure of both the unmodified and modified CNC samples, with the resulting spectra displayed in Figure 5.

In all the spectra obtained, a wide absorption peak was observed in the range of 3400–3300 cm⁻^1^, which corresponded to stretching and bending vibrations of hydroxyl groups (OH) within cellulose [26]. This signal indicates a significant amount of hydroxyl groups within the cellulose structure. The absorptions observed in the range of 2850–2950 cm⁻^1^ are associated with the C-H stretching vibrational modes present in the primary chain of cellulose, a characteristic representative of the polysaccharide form of CNC [27]. All samples exhibited an absorption band around 1640 cm⁻^1^, which corresponds to the bending mode of water molecules that are absorbed within the cellulose matrix [28]. The peaks observed at 1119 cm⁻^1^ and 1062 cm⁻^1^ are associated with the stretching vibrations of C-O bonds, thereby providing additional confirmation of the cellulose structure [29]. When CNCs were modified with 2,4-methylene diphenyl diisocyanate (MDI), several changes were recorded in the FTIR spectrum. A strong peak at 2270 cm⁻^1^ appears, characterizing the unreacted isocyanate (NCO) group of MDI. A urethane bond would be established between one of the two isocyanate groups and the hydroxyl groups present on the cellulose. This suggests that only one of the two isocyanate groups interacts with the hydroxyl groups on the cellulose to create a urethane bond, leaving the second NCO group unreacted. The peaks in the 3400–3300 cm⁻^1^ range, as well as the C-H and C-O signals, remain very similar to those of neat CNC, indicating that cellulose structure remains predominantly intact despite the modification with MDI. The other weak peak at approximately 1743 cm^−1^ is allocated to C=O groups from urethane bonds between the CNC and MDI [30]. The additional incorporation of castor oil in the CNC-MDI-CO sample results in further changes in the FTIR spectrum. A strong peak is detected at 2924 cm⁻^1^, which is likely attributed to the stretching of C-H bonds, while the peak observed at 2855 cm⁻^1^ corresponds to -CH_2_ groups, both from the long alkyl chains present in castor oil [31]. Additionally, a new, strong band appears at 1743 cm⁻^1^, which is related to carbonyl groups associated with the ester bonds present in castor oil. This peak distinguishes the CNC-MDI-CO sample from the CNC-MDI sample, in which a weaker absorption in this region is attributed to the urethane carbonyl groups [30].

### 3.2. XPS Analysis of CNC and Modified CNC Samples

XPS analysis of the elemental composition of neat CNC, CNC-MDI, and CNC-MDI-CO confirmed that isocyanate and castor oil modification on the surface of CNC has been successfully carried out. As depicted in Table 2, a high carbon and oxygen percentage is typical for cellulose, which consists mainly of carbon, hydrogen, and oxygen. The absence of nitrogen showed that native CNC lacks any nitrogenous compounds. While the carbon content increases from 64.1% to 80.8%, the oxygen content decreases from 34.7% to 12.7%, and nitrogen appears at 5.8%.

All these facts indicate that MDI has successfully interacted with the hydroxyl groups present on the CNC surface, causing the formation of nitrogen-containing isocyanate groups. This decrease in oxygen content agrees with the addition of organic isocyanate groups, which contain fewer oxygen atoms than the original cellulose.

Further modification of CNC-MDI increases the carbon content from 80.8% up to 85.1%, while reducing both oxygen, from 12.7% down to 12.4%, and nitrogen percentages, from 5.8% down to 2.5%. The changes in the elemental composition confirm the introduction of new chemical groups on the surface of the CNC, which may modify its properties for specific applications.

**Figure 6:**
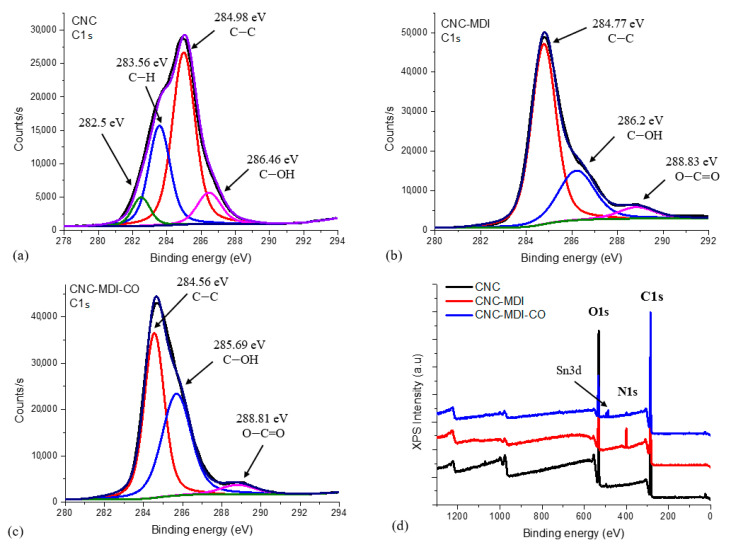
High-resolution C1s spectral deconvolution from XPS of (**a**) neat CNC, (**b**) CNC-MDI, and (**c**) CNC-MDI-CO, and (**d**) full range of XPS spectra of all samples.

The high-resolution deconvoluted C1s spectrum of CNC presented in Figure 6a displays peaks at 282.5 eV and 283.56 eV, which are likely attributed to C-H bonds. Additionally, the peak observed at 284.98 eV corresponds to C-C bonds, while the peak at 286.47 eV is associated with C-OH bonds [32]. In Figure 6b, the high-resolution deconvoluted C1s spectrum of CNC-MDI reveals peaks at 284.77 eV, corresponding to C-C bonds, at 286.2 eV, associated with C-OH bonds, and at 288.83 eV, which may indicate the presence of O-C=O ester bonds resulting from urethane formation [33].

In Figure 6c, the high-resolution deconvoluted C1s spectrum of CNC-MDI-CO displays peaks that are comparable to those of CNC-MDI; however, the most notable distinction in the XPS data is the intensity of the C-OH bonds. In the CNC-MDI sample, the C-OH bonds had an intensity of 12,596.94 counts/s, while in the CNC-MDI-CO sample the intensity was 22,078.8 counts/s due to additional OH groups from castor oil.

The XPS spectra of both CNC-MDI and CNC-MDI-CO clearly demonstrate the presence of C1s, O1s, and N1s peaks, whereas CNC shows only the peaks corresponding to C1s and O1s, as illustrated in Figure 6d. The Sn3d peak in CNC-MDI-CO represents the use of the catalyst dibutyltin dilaurate during its production. The deconvolution of the O1s peak presented in Figure 6d reveals three distinct peaks: one for CNC at 531.43 eV, which is associated with oxygen atoms in hydroxyl (OH) groups, and another for CNC-MDI at 532.81 eV, corresponding to oxygen atoms in carbonyl (C=O) groups [34], and CNC-MDI-CO at 533.02 eV, possibly corresponding to oxygen atoms in ester (C-O-C) or carboxylate (O-C=O) groups. The presence of the N1s peak in both CNC-MDI and CNC-MDI-CO indicates that the modification of MDI and CO on the surface of the CNC has been successfully conducted.

### 3.3. DSC Analysis of CNC and Modified CNC Samples

**Figure 7:**
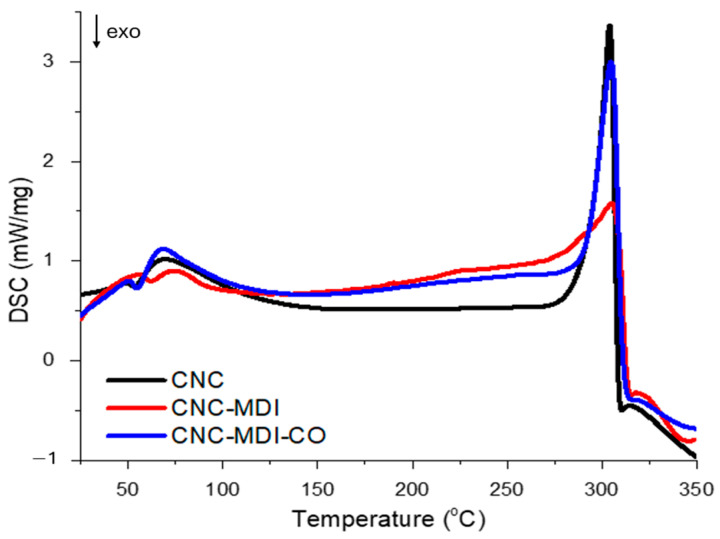
DSC profiles of CNC, CNC-MDI, and CNC-MDI-CO samples.

The significant thermal parameters derived from the DSC analysis of both the CNC and modified CNC samples are displayed in Table 3, and the corresponding thermal curves for the analyzed samples are illustrated in Figure 7.

Transition temperature, as obtained from the DSC spectrum, is one of the key factors that determines whether or not a material, after heating, will lose its structural integrity [35]. The thermal parameters of CNC indicate high energy absorption in both the thermal phases of CNC. First, the ΔH_1_ stage stayed at about 47.09 J/g at T_max1_ of 69.4° C and can be assumed to show evaporation of moisture or a slight decomposition process. In the second step, CNC absorbs a much greater energy of 263.9 J/g, whereas the temperature of peak degradation was 304.1 °C, which means that pure CNC has impressive thermal stability up to about 304 °C; at this value, it starts to degrade significantly.

In the CNC-MDI sample modified with isocyanate, energy absorption steeply decreases in both phases. ΔH_1_ goes down to 11.81 J/g, whereas T_max1_ slightly shifts to higher values, 74.5 °C, which could indicate some resistance from the modification to the first thermal event by the action of, probably, cross-linking effects. The second step, ΔH_2_, is lower, about 187.2 J/g, but T_max2_ is almost invariable at 305.2 °C; this means that the thermal degradation temperature is the same, but its general energy absorption is reduced compared to pure CNC. We note that the melting enthalpy, ΔH_1_, for the CNC-MDI-CO sample modified with both isocyanate and castor oil, increases to the value of 60.3 J/g, already larger than that of unmodified CNC, indicating a larger endothermal effect at the beginning of the sample. T_max1_ goes slightly down to 68.4 °C, with an onset of the thermal event a bit earlier. The second step is energy absorption of 244.3 J/g at T_max2_ of 304.8 °C, indicating the thermal stability of the material, though decomposition is more energetic, from which one can infer that treatment with castor oil increases the heat absorption characteristic of the material with no loss in its thermal stability.

### 3.4. TGA Analysis of CNC and Modified CNC Samples

**Figure 8:**
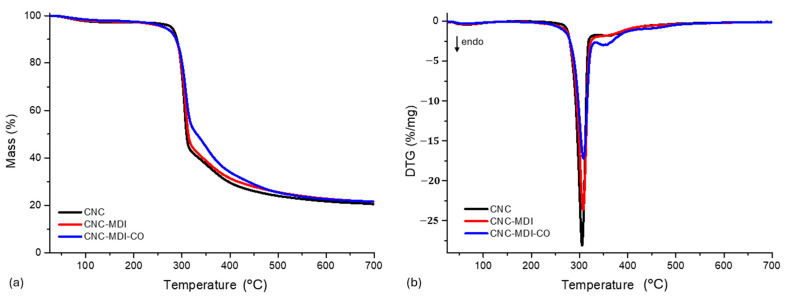
(**a**) TGA and (**b**) DTG curves for CNC and modified CNC samples.

Table 4 illustrates the percentage decomposition of CNC and modified CNC samples. Furthermore, the thermogravimetric analysis (TGA) curves, along with their respective derivative thermogravimetric (DTG) curves for each analyzed CNC and modified CNC sample, are displayed in Figure 8a,b.

The graphs obtained demonstrate the thermal degradation of the samples, and in fact they indicate that the addition of MDI and CO to CNC results in a slight alteration of the neat CNC. In particular, Td3%, Td5%, and Td10% in the CNC-MDI and CNC-MDI-CO composites are slightly lower compared with neat CNC, which means a slight loss in thermal stability. However, the discrepancy in those degradation temperatures remains within an acceptable range, and therefore it was demonstrated that surface modifications using MDI and CO do not substantially affect the thermal stability of composite materials. Furthermore, T_max_ values, obtained from the DTG curves, are almost identical for all samples, indicating that the maximum rate of thermal decomposition occurs within the same temperature range for both modified and unmodified CNC.

The overall stability in the percentage of mass loss across all samples further confirms that the incorporation of MDI/CO does not significantly influence the thermal degradation characteristics of the materials. These findings are in agreement with similar observations in other works [36], where the modifications performed on the surface do not cause strong changes in the degradation behavior of the CNC-based composites.

### 3.5. Contact Angle (CA) Analysis of CNC and Modified CNC Samples

**Figure 9:**
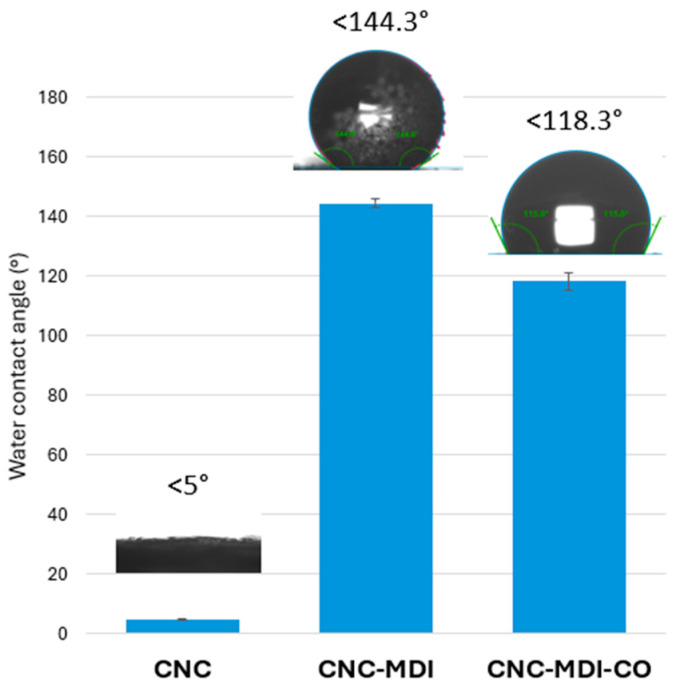
Water contact angle of CNC and modified CNC samples.

Figure 9 illustrates the water contact angle for both CNC and modified CNC samples. The measurement of the water contact angle serves as an effective method for evaluating the wettability of the surface of a material. The hydrophilic nature of CNC, attributed to its high concentration of hydroxyl groups, resulted in a contact angle of less than 5° for neat CNC [37]. Chemical modification of the CNC with MDI replaced the hydrophilic character of CNC with a more hydrophobic structure at 144.3° [38]. The reaction involves the substitution of hydrophilic hydroxyl groups with hydrophobic urethane and phenyl groups. The introduction of hydrophobic phenyl groups, along with a reduction in hydroxyl groups, results in an increased hydrophobicity of the surface.

With functionalization by castor oil, the hydrophobicity of CNC-MDI-CO decreased to 118.3°. The structure of castor oil contains hydroxyl groups, which are naturally hydrophilic, and this could help explain the phenomenon [39]. When CNC-MDI is reacting with castor oil, introducing more hydrophilic functional groups on the surface has led to a noticeable reduction in the overall hydrophobicity of the sample.

### 3.6. SEM Analysis of CNC and Modified CNC Samples

**Figure 10:**
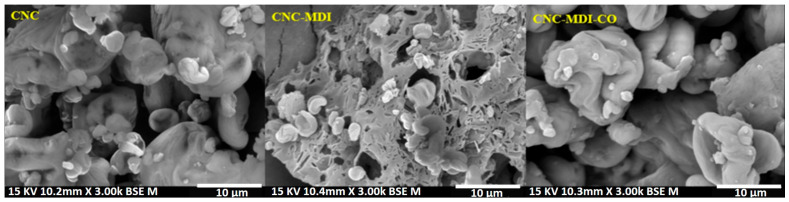
SEM samples of neat CNC, CNC-MDI and CNC-MDI-CO.

The morphology of CNC was examined using SEM, both before and after modification. Figure 10 illustrates the morphologies of the unmodified CNC as well as the modified CNC-MDI and CNC-MDI-CO samples.

Figure 10 shows the neat CNC morphology, where micrographs show a spherical shape, opposite to the rod-like structure. The CNCs have been observed to be clustered with a homogeneous distribution and moderate porosity. The neat CNC sample is found to have a smoother and more aggregated morphology.

The second picture represents CNC modified with MDI (isocyanate); its morphology is very different when compared with neat CNC. The structure of CNC-MDI has greater void spaces with more porous and less compacted clusters than the neat CNC. The possible reason for the generation of these voids includes the interaction of CNC with MDI, which might indicate surface roughening and increased porosity upon modification. It is, however, appreciably more irregular, which would support that the MDI has interacted with both the CNC dispersion and cross-linking processes at the surface, yielding a structure that is less compact and more open.

From the last image, it can also be observed that the CNC-MDI-CO sample has a smooth surface, while it has kept a relatively porous structure. This might be due to the action of castor oil acting with the CNC-MDI matrix, initiating the development of a more elastic and flexible network.

The presence of castor oil seems to contribute to a slightly more compact morphology than that found in the CNC-MDI sample. However, SEM images reveal that neat CNCs tend to aggregate into spherical structures with dimensions of a few micrometers during the drying process, rather than maintaining their rod-like morphology. This aggregation likely occurs due to capillary forces during evaporation, emphasizing the need to optimize drying techniques. Methods such as lyophilic drying or sequential solvent exchange (e.g., from water to acetone and then to toluene) could be explored to minimize aggregation and enhance the dispersion of CNCs during the reaction with MDI. Furthermore, the treatments with components such as MDI and castor oil improve the porosity, surface roughness, and flexibility of CNCs and their related materials [40,41]. The improved mechanical properties from these modifications include enhanced elasticity and network flexibility, However, the morphology exhibits varied structures, which in turn influence the overall dispersion of the material. These morphological changes reflect the chemical modifications of MDI and castor oil, which influence dispersion, surface roughness, and generally the whole structure of the CNC samples.

### 3.7. FTIR Analysis of CNC- PLA Nanocomposites

**Figure 11:**
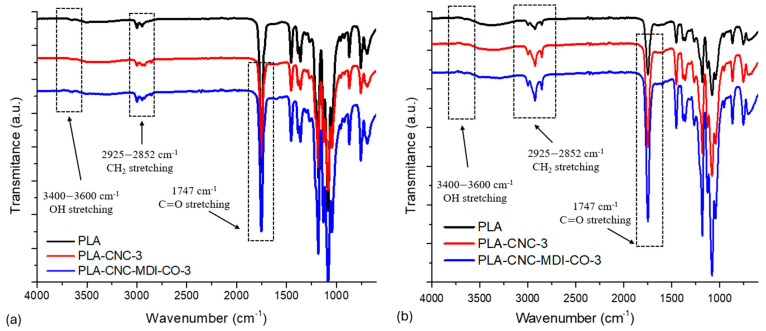
FTIR spectra of neat PLA and modified PLA composites that have been produced through (**a**) solvent casting (SC) and (**b**) Pickering emulsion (PE).

The FTIR spectra presented in Figure 11 illustrate the trends identified for the PLA, PLA-CNC, and PLA-CNC-MDI-CO samples, which were produced using the SC and PE methods. The data indicates a notable distinction among all samples regarding molecular interactions and the dispersion of additives. In both preparation techniques, the distinctive C=O stretching around 1700 cm⁻^1^ and the C-O-C stretching in the range of 1000–1200 cm⁻^1^ become more pronounced with the introduction of CNC and the functionalization process involving MDI and castor oil, proving the successful incorporation of these molecules. However, much sharper and better-defined peaks, especially at these regions, are observed in the Pickering emulsion (PE) samples when compared to broader peaks represented in the solvent-casting (SC) samples. These sharper peaks in PE suggest an improved molecular organization and superior dispersion of CNC and MDI-CO within the PLA matrix, which could relate to better emulsification and stronger interfacial interactions between the polymer and additives. On the other hand, the broader peaks in the SC samples suggest a more non-homogeneous distribution that might arise from either agglomeration or incomplete interaction of the additives with the polymer matrix. In certain studies, the spectrum of PLA/CNC composites showed no significant differences compared to pure PLA, indicating that there are no strong interactions between the CNC and PLA chains [42]. However, the presence of MDI and castor oil seems to improve interactions, especially in the PE method. This comparison underlines that the PE method provides higher chemical interactions and homogeneity for the composite structure compared to SC.

### 3.8. DSC Analysis of PLA-CNC Nanocomposites

**Figure 12:**
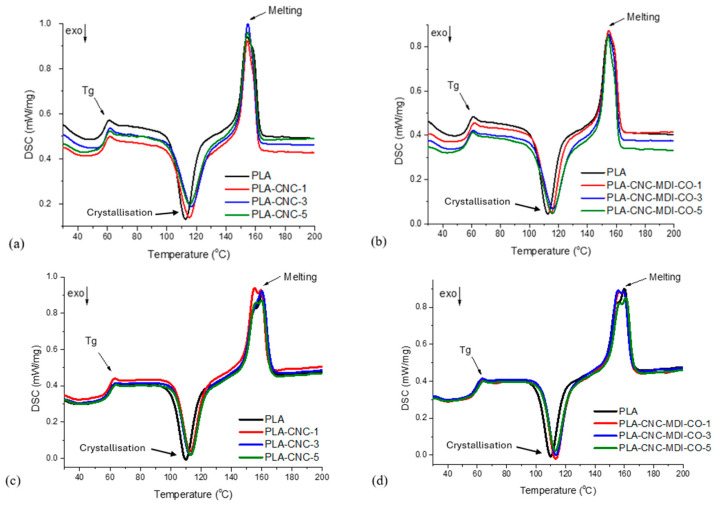
DSC profiles of (**a**) PLA-unmodified CNC samples (SC), (**b**) PLA-modified CNC samples (SC), (**c**) PLA-unmodified CNC samples (PE), and(**d**) PLA-modified CNC samples (PE).

The thermal characteristics obtained from the differential scanning calorimetry (DSC) analysis for PLA-CNC nanocomposite samples through solvent-casting and Pickering emulsion techniques are shown in Table 5. The transition temperatures (T_c_) and enthalpy changes (ΔH_c_) associated with crystallization were calculated during the cooling phase. Subsequently, in the second heating cycle, the glass transition temperature (T_g_), cold crystallization temperature (T_cc_), melting temperature (T_m_), cold crystallization enthalpy (ΔH_cc_), and melting enthalpy (ΔH_m_) were assessed. Furthermore, Figure 12 displays the DSC profiles of the modified and unmodified PLA-CNC samples.

The degree of crystallinity in a material can be determined by using the following formula:

$$Crystalinity,\chi \;\left(\%\right)=\frac{\Delta H_{m}}{\Delta H_{m},0}\;\times \;100\;$$

ΔH_m_ represents the melting enthalpy of the sample (J/g).

The theoretical melting enthalpy of 100% crystalline PLA is denoted as ΔH_m_,0 and is measured in joules per gram (J/g).

Since PLA is partially crystalline, the theoretical melting enthalpy (ΔH_m_,0) can be determined by assuming complete crystallinity using the literature value. It is estimated that about 93 J/g of heat energy was required to make fully crystalized PLA melt [43].

Table 4 indicates that there were no notable alterations concerning the glass transition temperature of both PLA and PLA/CNC nanocomposites [44].

Thermal analysis findings suggested that the Pickering emulsion technique significantly enhanced crystallinity and thermal properties in all samples when compared to the solvent-casting method. During the emulsification process, the nanoparticles function as nucleating agents, thereby facilitating enhanced crystallization during these phases; therefore, better and more ordered crystallization can take place in Pickering emulsion samples. This is in very good agreement with literature data suggesting that nanoparticle-stabilized systems promote more nucleated sites, therefore resulting in enhanced crystallinity in biodegradable polymers such as PLA [45]. On the other hand, solvent-casting samples display a lower crystallinity, apparently because of the more reduced evaporation rate and the absence of any nucleation agent, similarly to what has been reported for similar studies using a solvent-casting technique [46].

These advantages of the PE method are further highlighted by the differences in melting enthalpy, ΔH, and cold crystallization enthalpy, ΔH_cc_. Indeed, samples obtained by PE always show an increased ΔH_m_, indicating the presence of a larger number of thermally stable and ordered crystalline fractions. In contrast, the enhanced ΔH_cc_ values present in solvent casting. The samples indicate more amorphous regions requiring extra heat to crystallize from the heated sample, which is consistent with previous studies showing that solvent casting results in lower levels of crystallization and more amorphous phases being preserved [47]. The T_cc_ for cold crystallization is higher in samples prepared by solvent casting, that is, a greater extent of disordered structures crystallized at higher temperatures. The shift of the melting temperatures in PE samples to higher values proves that crystalline regions are thermally more stable. It is highly likely that the improved and more uniform growth of crystals can be assigned to the dispersion of particles.

Works on Pickering emulsions have also reported an increase in the stability of crystalline structures by such systems through the improvement in particle dispersion [48]. While the glass transition temperature (T_g_) remains identical in the two methods, indicating lesser influence on the amorphous regions, the resultant increased crystallinity and heat resistance make the PE technique superior in bringing out more significant thermal properties in PLA-based materials.

The presence of castor oil may result in the plasticization effect, explaining the thermal behavior of modified CNC. With extended aliphatic chains, the castor oil would easily diffuse into the PLA molecular chains. Such an observation has also been recorded in the literature while blending PLA with various plasticizers [49,50]. Although PLA was prepared with the incorporation of CNC or MDI-CO using different methods, the thermal properties, such as crystallinity, melting behavior, and glass transition temperature, did not vary significantly and remained basically constant

### 3.9. TGA Analysis of PLA-CNC Nanocomposites

**Figure 13:**
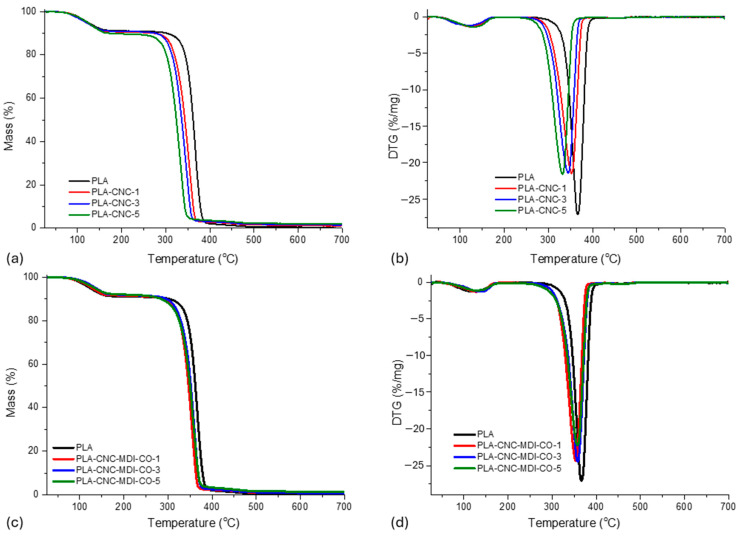
(**a**–**d**) Thermogravimetric analysis (TGA) and derivative thermogravimetric (DTG) curves for polylactic acid (PLA) and PLA composites produced via solvent casting.

The TGA curves as well as the derived thermogravimetric (DTG) curves of each analyzed CNC/PLA-CNC and PLA-modified CNC sample produced via the solvent-casting method are illustrated in Figure 13a–d and in Table 6.

The TGA data for solvent-casting samples indicate that the weight loss of PLA occurs within the temperature range of 100–360 °C. This phenomenon is assigned to thermal degradation resulting from transesterification reactions that destroy the integrity of the main structure of PLA [51]. It was interesting to note, however, that the decomposition of the neat PLA started at 124 °C (Td5%), whereas its maximum degradation rate was 367 °C. The addition of CNC to PLA seems to affect its stand in thermal degradation. Introducing CNC slightly lowers the temperatures at which recognizing weight losses occur (Td5%, Td10%) compared to neat PLA. Among the CNC-modified samples, PLA-CNC-3 displayed the lowest thermal stability, as reflected from the lowest Td5% value depicted. The T_max_ value obtained from the DTG analysis of the modified samples is found to be lower than that of the unmodified PLA, suggesting alterations in the degradation kinetics. Similar results were reported in Luzi et al. [52]. Although there are some differences, the mass loss percentages of the modified samples are relatively similar and slightly lower than that of neat PLA. This indicates that the incorporation of CNC does not substantially alter the degradation behavior trend of the composite materials.

Incorporating 2,4-MDI diisocyanate and castor oil into PLA-CNC-MDI-CO slightly modifies the thermal degradation behavior compared with neat PLA. All in all, the modified samples exhibit similar or slightly lower Td5% and Td10% temperatures compared to neat PLA, indicating a decrease in the thermal stability of these samples. PLA-CNC-MDI-CO samples generally seemed to have a higher T_max_, meaning better thermal resistance, compared with its derivatives with just CNC but lower than neat PLA.

**Figure 14:**
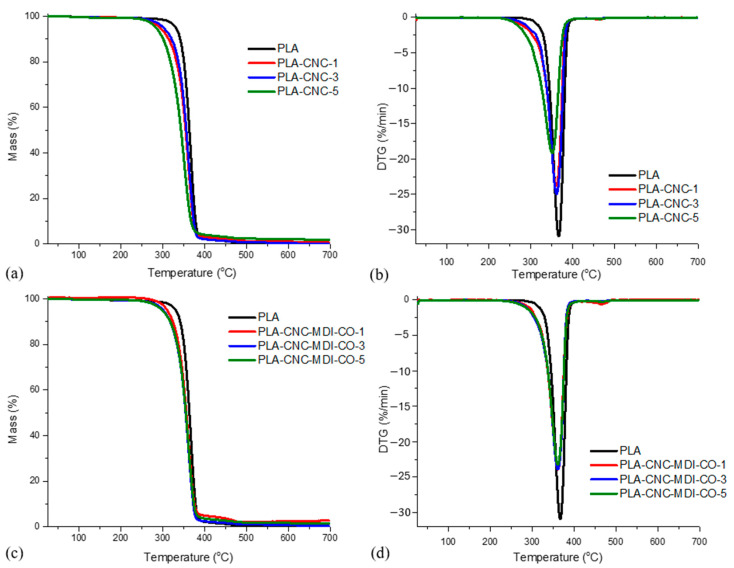
(**a**–**d**) Thermogravimetric analysis (TGA) and derivative thermogravimetric (DTG) curves for polylactic acid (PLA) and PLA composites produced via Pickering emulsion (PE).

The mass loss of the modified samples is also comparable to neat PLA or slightly lower, which suggests no significant effect on adding MDI and CO onto the composite material in relation to its overall degradation behavior. The TGA curves and the derived thermogravimetric curves (DTG) of each analyzed sample CNC/PLA-CNC and PLA-modified CNC through the Pickering method are presented in Figure 14a–d.

TGA analysis of the Pickering emulsion samples shows that neat PLA exhibits good thermal stability with relatively high values of Td5%, Td10%, and T_max_. Mass is nearly totally lost, showing intense decomposition. The breakdown of a PLA matrix would include the breaking of functional groups such as C-C, C-O, or C=O bonds. The PLA shows weight loss within a temperature range of 300–372 °C, assigned to thermal degradation due to transesterification reactions that cleave the backbone structure of PLA [53].

With the addition of 1% CNC, Td5%, Td10%, and T_max_ are lower than neat PLA, a reflection of reduced thermal stability. The trend tends to follow at the 3% of CNC that thermal stability is still below that of neat PLA but higher than that of PLA-CNC-1, therefore indicating partial recovery in thermal properties. Further decrease in thermal stability at 5% CNC sample suggests that higher content of CNC negatively impacts thermal properties of the composite. The addition of MDI-CO with 1% CNC succeeded in enhancing thermal stability close to neat PLA, while MDI-CO at 3% CNC further increased thermal stability over PLA-CNC-3 but remained well below the neat PLA value. At 5%, CNC grafted with MDI-CO showed a moderate enhancement in thermal properties over PLA-CNC-5 but was lower than neat PLA.

The thermal stability of neat PLA is the most significant, exhibiting nearly complete decomposition. The addition of CNC generally reduces thermal stability, although further loading of CNC leads to a reduction in Td5%, Td10%, and T_max_ values. The addition of MDI-CO improves the thermal stability of PLA-CNC composites. Consequently, while CNC tends to reduce the thermal stability of PLA composites, the addition of MDI-CO seems to mitigate this effect and enhances the properties towards the neat PLA matrix. Similar observations have been obtained for several studies [54,55]. The mass loss is generally high in all samples; however, the Pickering emulsion method has presented lower mass loss for PLA-CNC-MDI-CO, showing improved stability. Therefore, the Pickering emulsion method shows potential for the development of CNC-PLA composites that exhibit improved thermal stability with the incorporation of MDI-CO. The Pickering emulsion technique seems to impart more enhanced thermal stability into the composites than the solvent-casting method.

### 3.10. Mechanical Tests of PLA-CNC Nanocomposites

The tensile properties and modulus of neat PLA, as well as the composites PLA/CNC and PLA/modified CNC, which were prepared using the solvent-casting and Pickering emulsion techniques at various CNC contents, are presented in Table 7.

The PLA matrix clearly shows that as the content of CNC and modified CNC increases, the tensile strength of the composite material tends to decrease. Additionally, the tensile strength of pure PLA compared to PLA mixed with additives can vary due to specific formulations or processing methods. Normally, pure PLA has around 45 to 65 MPa in tensile strength [42]. The introduction of additives into PLA resulted in a noticeable decline in tensile strength. Specifically, the tensile strength decreased from 20.3 MPa to 13.5 MPa with the addition of CNC and modified CNC to the PLA matrix [56].

The trends of the mechanical properties of PLA-based composites are different in terms of synthesis methods and the nature of additives, particularly in the case of such aspects as stiffness–ductility–strength. The observation to be noted herein is that the addition of cellulose nanocrystals, MDI, and castor oil had different shifts in the stiffness–flexibility balance, and the overall mechanical performance of PLA has been improved to some extent. The overall trend of incorporating CNC typically improves the stiffness of PLA because of its reinforcing properties; however, this is always at the price of reduced flexibility and tensile strength. In this case, in the solvent-casting technique the PLA-CNC sample shows a moderate rise in the stiffness of, for example, PLA-CNC-3 with 860.0 MPa, which incorporates considerable loss regarding flexibility when compared to pure PLA, which tended to develop a maximum tensile strain of up to 23.5%. Similarly, in the Pickering emulsion method, the incorporation of CNC enhanced stiffness up to a certain point, after which it began to decrease. While PLA-CNC-1 had 2825.2 MPa, for instance, the stiffness of PLA-CNC-5 went down to 2635.6 MPa. The main cause for this observation is that, generally, CNC forms rigid networks that hinder matrix flexibility and therefore show a sharp decline in ductility

Also, the addition of MDI and CO to composites increases flexibility properties and tensile strength up to a partial recovery. For solvent-cast systems, they showed a significant increase of stiffness with the addition of MDI and CO to PLA-CNC (PLA-CNC-MDI-CO samples) in comparison to PLA-CNC alone (e.g., 1260.0 MPa for PLA-CNC-MDI-CO-3), followed by slight improvements in tensile stress and strain in some cases (e.g., PLA-CNC-MDI-CO-1 achieved 1.87% of strain and 30.8 MPa stress in Pickering emulsion). Nevertheless, such modifications did not highly recover the lost flexibility similar to that in PLA-CNC where both PLA-CNC-MDI-CO-3 and PLA-CNC-MDI-CO-5 provided lower tensile strain (5%, 5.3%) and lower tensile stress due to potential challenges of dispersing CNC, MDI, and CO homogeneously. Additionally, such poor dispersion may cause weak interfacial adhesion, and in turn the mechanical strength of the resulting composite is impaired.

MDI and CO could produce a good stiffening effect in the solvent-casting samples (e.g., PLA-CNC-MDI-CO-3 with 1260.0 MPa) along with a little higher flexibility compared to CNC-only composites. The tendency of Young’s modulus decreases slightly with respect to pure PLA (i.e., PLA-CNC-MDI-CO samples show regular lower Young’s modulus in all cases), showing that the mix of MDI and CO increases flexibility and stiffness similarly. This implies that the CNC acts as a filler, while the MDI and CO are plasticizers, hence balancing stiffness with flexibility.

The general reduction in flexibility and tensile strength observed in CNC-based composites can be attributed to inadequate dispersion and insufficient interfacial bonding between the polymer matrix and the filler [57]. The addition of MDI and CO helps to reduce this to some extent by forming urethane bonds (in the case of MDI) and introducing alkyl chains (from CO), but these modifications are not sufficient to entirely overcome the brittleness caused by CNC.

Among the synthesized composites, PLA-CNC-MDI-CO-1 presents an optimal balance of stiffness, 2325.6 MPa, tensile strain, 1.87%, and tensile strength, 30.8 MPa. Thus, PLA-CNC-MDI-CO-1 is the most promising material intended for packaging related to medium level(s) of flexibility and strength. The addition of MDI and CO decreases the rigidity levels of CNC for a versatile, economical, and greener alternative to pure PLA, specifically for semi-rigid or flexible packaging.

In a different study [58], PLA was mixed with 5–20 wt % of natural castor oil and 15 wt % of modified castor oil to improve its mechanical properties. The results indicate noticeable flexibility and heat resistance improvements, making it a suitable option for packaging applications requiring flexibility. However, this research provides numerous advantages, particularly for packaging that needs a mix of durability, flexibility, and heat resistance.

There are many important factors when considering the best candidate for packaging materials among the given samples: the balance between mechanical properties (stiffness, flexibility, and strength), environmental impact, and cost. Comparing the two methods, it was found that the Pickering emulsion method gave better general results than the solvent-casting method. The improved dispersion and distribution of CNC-MDI and CO with PLA, achieved through the Pickering emulsion method, leads to enhanced interaction with the PLA matrix. This results in a greater effectiveness of the modified CNC [59].

### 3.11. Contact Angle (CA) Analysis of PLA-CNC Nanocomposites

**Figure 15:**
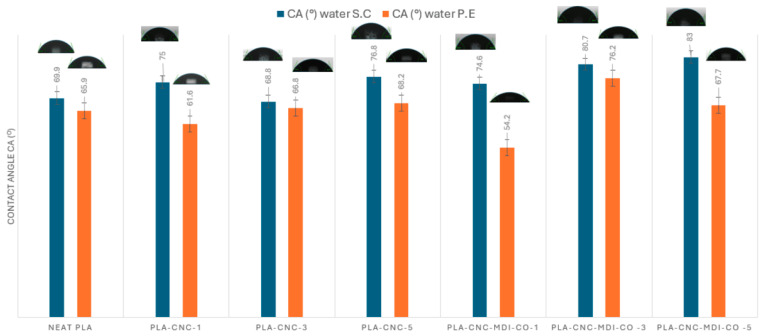
Water contact angle of neat PLA, and PLA composites through solvent-casting and Pickering emulsion methods.

Hydrophobicity provides a better water resistance, lower moisture permeability, and high mechanical strength for food packaging materials [60]. Different variations and combinations of CNC, MDI, and castor oil can change the surface roughness and texture. The polar groups interact differently on the surface of the materials depending on their concentrations, altering the surface energy and wetting properties of the samples, and affecting the contact angles. To assess and compare the wettability of each sample, we measured the water contact angles (CA) on the surfaces of all samples, as illustrated in Figure 15.

Neat PLA generally has a lower contact angle (69.9°) than the PLA composites but also has some hydrophobic characteristics. Adding CNC and modified CNC samples to the PLA matrix has increased hydrophobicity, raising the contact angle. The presence of castor oil in the CNC-MDI-CO composites could contribute to increased hydrophobicity due to its hydrophobic nature.

The addition of modified CNC led to an increase in the water contact angle across all nanocomposites as expected. Previous studies have indicated that a crosslinking structure and reduced surface tension can enhance hydrophobic characteristics [61].

With the exception of the 1% of CNC and modified CNC samples that utilized the CO-modified CNC samples in PLA composites, the water contact angle (WCA) exhibited an increase from 68.9° in 3% PLA-CNC to 80.7° in 3% PLA-CNC-MDI-CO, and from 76.8° in 5% PLA-CNC to 83.0° in 5% PLA-CNC-MDI-CO. This can be attributed to interactions between castor oil and PLA, which may result in a more hydrophobic surface on the surface of composites. This is probably due to the presence of long side chains in castor oil, which may lead to an increase in intermolecular bonds or crosslinking. This, in turn, diminishes the accessibility of hydrophilic functional groups on the surface of PLA [62].

Neat PLA obtained by Pickering emulsion has a contact angle (65.9°). Adding 1% CNC made the surface more hydrophilic (61.6°); this could be attributed to the hydrophilic nature of cellulose nanocrystals. At 3% and 5% CNC concentrations, they form a more tightly organized surface, which enhances hydrophobicity.

In the PLA-CNC-MDI-CO-1 sample, a significant reduction in the contact angle (54.2°) was observed. The observed phenomenon may result from the incorporation of MDI and castor oil, which enhances the surface’s hydrophilicity. This effect is likely attributed to the polar groups present in MDI and the hydroxyl groups found in castor oil. However, PLA-CNC-MDI-CO-3 showed more hydrophobic surfaces (76.2°). This could be due to the higher concentration of MDI that creates a more hydrophobic network or changes in surface morphology. In the PLA-CNC-MDI-CO-5 sample, the effect of additional modified nanocellulose balances out, resulting in a moderately hydrophobic surface (67.7°).

The solvent-casting method generally produces more hydrophobic surfaces compared to Pickering emulsion, especially at higher concentrations of CNC and with the addition of MDI and castor oil. On the other hand, the Pickering emulsion method makes the surfaces more hydrophilic, especially at lower concentrations of CNC and with the addition of MDI and castor oil at 1%. However, it also produces highly hydrophobic surfaces at 3% MDI and castor oil concentration. The two methods likely result in different surface structures, affecting wettability.

### 3.12. SEM Analysis of PLA-CNC Nanocomposites

**Figure 16:**
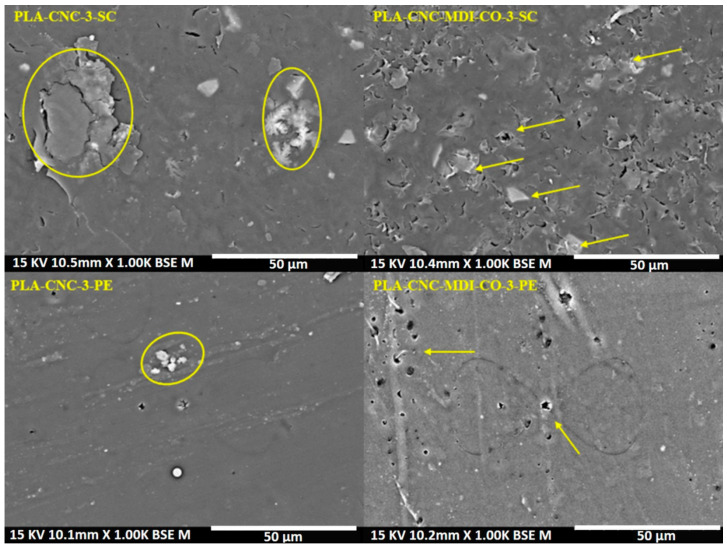
SEM images of PLA-CNC and PLA-modified CNC samples, which were prepared using solvent-casting (SC) and Pickering emulsion (PE) methods, with a concentration of 3% by mass.

The morphology of PLA composites was examined using scanning electron microscopy (SEM). Figure 16 illustrates the morphology of the PLA-CNC samples and the PLA-modified CNC samples, which contain 3% by mass. The micrographs present a remarkable difference in the surface morphologies due to the different preparation methods of PLA-CNC-3-SC and PLA-CNC-3-PE samples. The solvent-casting method has produced a relatively rough surface with some visible agglomerates (indicated by the yellow circles and arrows) for the PLA-CNC-3-SC sample, which could signify a nonuniform distribution of cellulose nanocrystals and their high clustering [9]. While the Pickering emulsion method has produced an overall smoother surface of the PLA-CNC-3-PE sample, this points toward the relatively more homogeneous distribution of nanocrystals, which can contribute to a better barrier performance and higher mechanical strength for possible further applications [63].

Similarly, the PLA-CNC-MDI-CO-3-SC sample prepared by the solvent-casting method clearly indicates a more heterogeneous surface with bigger, irregular features, hinting at the idea that the addition of Isocyanate and castor oil results in a complex microstructure. Conversely, the surface features of the PLA-CNC-MDI-CO-3-PE sample, formed by the Pickering emulsion method, are smoother, which is typical for this emulsion methodology, as well as introducing some irregular features from the added components. These differences clearly reflect how the choice of the preparation method can be crucial, together with the inclusion of specific additives, in substantially altering the microstructure and, consequently, the material properties of PLA composites.

## 4. Conclusions

The work presented here was divided into two parts and began with the modification of CNC using MDI and castor oil, which greatly altered its properties. Grafting with MDI improved compatibility and interfacial adhesion, as can be derived from FTIR and XPS analyses. However, the aggregation of CNCs during the preparation and drying processes resulted in the formation of large microparticles instead of nanoparticles, as evidenced by SEM. This aggregation was a significant factor in the reduced mechanical properties observed in some samples, particularly those prepared by the SC method. The results of the contact angle tests indicated that incorporating castor oil resulted in a reduction of hydrophobic properties. TGA exhibited acceptable thermal stability, while SEM studies revealed increased porosity and surface roughening, hence enhancing the flexibility and mechanical performance as well. Two preparation methods were compared in the second part: solvent casting (SC) vs. Pickering emulsion (PE). In both cases, the PE method proved superior and resulted in an improved dispersion of CNC within the PLA matrix. This improvement was supported through both FTIR and SEM analyses. For example, sharper FTIR peaks for the PE-based samples are indicative of strong interfacial bonding, whereas the broader peaks found in SC-based samples were representative of less homogeneous dispersions. Despite the aggregation challenges, the findings from the thermal analysis indicated that the PE-based samples exhibited a 15% increase in crystallinity, along with enhanced thermal stability, degrading at 335 °C, in contrast to the SC-based samples, which degraded at 320 °C. because of relatively more ordered crystalline regions provided by the emulsification process after CNCs acted as nucleating agents. In the case of the SC-based samples, rather lower crystallinity was recorded due to the fact that the CNCs aggregated. The mechanical test demonstrated that, under poor dispersions, all the PE-based samples exhibited enhanced tensile strength and flexibility: up to 20% in tensile strength compared to SC-based samples. Notably, PLA-CNC-MDI-CO-1, from PE, reached an optimal compromise between rigidity, 2325.6 MPa, ductility, 1.87%, and tensile strength, 30.8 MPa; these samples were ideal choices for applications that require reasonable strength with moderate flexibility. The contact angle test results confirmed that the hydrophobicity was greater in PE-based samples compared with others, due to the better control over the dispersion of CNC agglomerates and consequently surface morphology. Therefore, the PE method proved to be more successful in enhancing the molecular, thermal, and mechanical characteristics of CNC-PLA composites. So, the PE method could be one of the most prospective ways to develop sustainable and high-performance biodegradable packing materials. This study highlights the persistent challenge of CNC aggregation during the drying and preparation stages. Preventing CNC aggregation during their preparation for modification with MDI is crucial to further improving dispersion and mechanical performance. Future research should focus on optimizing CNC, MDI, and castor oil concentrations and exploring strategies to minimize aggregation, such as alternative drying or stabilization techniques followed by scale-up processes for industrial application.

## Figures and Tables

**Table 1:** Mechanical properties of biopolymer-based and petrochemical-based plastics.

Material	Young’s Modulus (MPa)	Tensile Strength (MPa)	Elongation at Break (%)	Reference
Poly(lactic acid) (PLA)	3600	49.6	2.4	[2]
Poly(hydroxyalkanoate)s (PHA)s	200–3500	17–40	5–680	[3]
Low-density polyethylene (LDPE)	210	11,000	190	[4]
High-density polyethylene (HDPE)	911	20.3	380	[4]
Poly(ethylene tetraphthalate) (PET)	2700	55	130	[4]

**Table 2:** Elemental composition of CNC and modified CNC samples.

Sample	C at. %	O at. %	N at. %
Neat CNC	64.1	34.7	-
CNC-MDI	80.8	12.7	5.8
CNC-MDI-CO	85.1	12.4	2.5

**Table 3:** Thermal parameters of samples for CNC and modified CNC samples.

Samples	ΔH_1_ (J/g)	T_max1_ (°C)	ΔH_2_ (J/g)	T_max2_ (°C)
Neat CNC	47.09	69.4	263.9	304.1
CNC-MDI	11.81	74.5	187.2	305.2
CNC-MDI-CO	60.3	68.4	244.3	304.8

**Table 4:** Percentage decomposition of CNC and modified CNC samples.

Samples	T_d3%_ (°C)	T_d5%_ (°C)	T_d10%_ (°C)	T_max_ (°C) from DTG	Mass Loss (%)
Neat CNC	233	279	290	305	79.6
CNC-MDI	218	264	288	308	78.6
CNC-MDI-CO	228	265	288	308	78.5

**Table 5:** Thermal parameters of samples for solvent-casting (SC) and Pickering emulsion (PE) methods.

Sample	ΔH_cc_ (J/g)		T_cc_ (°C)		ΔH_m_ (J/g)		T_m_ (°C)		T_g_ (°C)		χ (%)	
	SC	PE	SC	PE	SC	PE	SC	PE	SC	PE	SC	PE
PLA	32.1	29.05	112.8	110.0	29.9	34.23	154.0	160.1	57.8	57.2	32.2	36.8
PLA-CNC-1	33.1	30.15	115.3	113.1	26.7	34.44	154.5	159.5	58.5	58.1	28.7	37.0
PLA-CNC-3	29.9	30.78	116.3	113.0	28.1	36.54	154.7	160.1	58.2	58.4	30.2	39.3
PLA-CNC-5	30.8	28.27	115.5	113.3	28.6	33.29	154.5	160.0	58.3	58.9	30.8	35.8
PLA-CNC-MDI-CO-1	30.8	29.40	115.1	113.4	29.3	35.27	154.6	159.5	58.6	57.6	31.5	37.9
PLA-CNC-MDI-CO-3	30.9	32.09	116.2	113.6	28.4	34.53	154.8	159.4	57.2	57.9	30.5	37.1
PLA-CNC-MDI-CO-5	30.6	29.77	115.8	113.3	27.8	32.58	154.4	161.1	57.6	58.2	29.9	35.0

**Table 6:** Percentage decomposition of neat PLA and PLA-CNC composites through solvent casting and Pickering emulsion.

Sample	T_d5%_ (°C)		T_d10%_ (°C)		T_max_ (°C) from DTG		Mass Loss (%)	
	SC	PE	SC	PE	SC	PE	SC	PE
PLA	124	331.3	304	341.8	367	367.4	99.95	99.82
PLA-CNC-1	122	293.5	275	313.8	353	361.9	98.94	99.27
PLA-CNC-3	120	301.6	277	321.4	345	361.8	98.56	99.98
PLA-CNC-5	123	284.1	274	301.0	332	350.8	98.06	98.14
PLA-CNC-MDI-CO-1	128	310.8	290	324.2	354	361.6	99.01	97.26
PLA-CNC-MDI-CO-3	136	301.4	297	317.7	358	361.5	99.41	99.64
PLA-CNC-MDI-CO-5	134	299.1	285	317.8	358	361.4	98.70	98.55

**Table 7:** Mechanical tests of colvent-casting and Pickering emulsion samples.

Sample	Solvent Casting			Pickering Emulsion		
	Young Modulus (MPa)	Tensile Strain (%)	Tensile Stress (MPa)	Young Modulus (MPa)	Tensile Strain (%)	Tensile Stress (MPa)
PLA	1340.0 ± 21.6	23.5 ± 0.6	20.3 ± 0.3	2764.0 ± 53.2	3.97 ± 0.65	49.7 ± 1.2
PLA-CNC-1	1034.6 ± 30.7	17.6 ± 0.5	20.1 ± 0.5	2825.2 ± 78.0	1.45 ± 0.12	35.2 ± 3.8
PLA-CNC-3	860.0 ± 27.7	11.0 ± 0.6	18.6 ± 0.5	2710.8 ± 87.5	1.12 ± 0.10	27.0 ± 2.2
PLA-CNC-5	906.7 ± 46.8	9.8 ± 0.6	18.4 ± 0.5	2635.6 ± 76.9	0.82 ± 0.14	20.8 ± 3.8
PLA-CNC-MDI-CO-1	1082.7 ± 20.6	8.4 ± 0.5	20.6 ± 0.3	2325.6 ± 29.3	1.87± 0.14	30.8 ± 1.2
PLA-CNC-MDI-CO-3	1260.0 ± 20.5	5.0 ± 0.4	18.1 ± 0.3	2452.0 ± 59.6	1.05 ± 0.16	23.7 ± 3.3
PLA-CNC-MDI-CO-5	1062.7 ± 18.3	5.3 ± 0.3	13.5 ± 0.2	2338.0 ± 79.9	0.78 ± 0.13	17.6 ± 3.1

## Data Availability

The original contributions presented in this study are included in the article. Further inquiries can be directed to the corresponding author.
